# Shelf-Life Evaluation and Lyophilization of PBCA-Based Polymeric Microbubbles

**DOI:** 10.3390/pharmaceutics11090433

**Published:** 2019-08-26

**Authors:** Tarun Ojha, Vertika Pathak, Natascha Drude, Marek Weiler, Dirk Rommel, Stephan Rütten, Bertram Geinitz, Mies J. van Steenbergen, Gert Storm, Fabian Kiessling, Twan Lammers

**Affiliations:** 1 Department of Pharmaceutics, Utrecht Institute for Pharmaceutical Sciences (UIPS), Utrecht University, 3584 CG Utrecht, The Netherlands; 2 Institute for Experimental Molecular Imaging, Faculty of Medicine, RWTH Aachen University, 52074 Aachen, Germany; 3 DWI—Leibniz Institute for Interactive Materials, Aachen, Germany AVT—Biochemical Engineering, RWTH Aachen University, 52074 Aachen, Germany; 4 Electron Microscope Facility, University Hospital RWTH, RWTH Aachen University, 52074 Aachen, Germany; 5 AVT—Biochemical Engineering, RWTH Aachen University, 52074 Aachen, Germany; 6 Department of Targeted Therapeutics, MIRA Institute for Biomedical Technology and Technical Medicine, University of Twente, 7500 AE Enschede, The Netherlands

**Keywords:** PBCA, microbubbles, ultrasound, lyophilization, storage stability, drug retention

## Abstract

Poly(*n*-butyl cyanoacrylate) microbubbles (PBCA-MB) are extensively employed for functional and molecular ultrasound (US) imaging, as well as for US-mediated drug delivery. To facilitate the use of PBCA-MB as a commercial platform for biomedical applications, it is important to systematically study and improve their stability and shelf-life. In this context, lyophilization (freeze drying) is widely used to increase shelf-life and promote product development. Here, we set out to analyze the stability of standard and rhodamine-B loaded PBCA-MB at three different temperatures (4 °C, 25 °C, and 37 °C), for a period of time of up to 20 weeks. In addition, using sucrose, glucose, polyvinylpyrrolidone (PVP), and polyethylene glycol (PEG) as cryoprotectants, we investigated if PBCA-MB can be lyophilized without affecting their size, concentration, US signal generation properties, and dye retention. Stability assessment showed that PBCA-MB remain largely intact for three and four weeks at 4 °C and 25 °C, respectively, while they disintegrate within one to two weeks at 37 °C, thereby compromising their acoustic properties. Lyophilization analyses demonstrated that PBCA-MB can be efficiently freeze-dried with 5% sucrose and 5% PVP, without changing their size, concentration, and US signal generation properties. Experiments involving rhodamine-B loaded MB indicated that significant dye leakage from the polymeric shell takes place within two to four weeks in case of non-lyophilized PBCA-MB. Lyophilization of rhodamine-loaded PBCA-MB with sucrose and PVP showed that the presence of the dye does not affect the efficiency of freeze-drying, and that the dye is efficiently retained upon MB lyophilization. These findings contribute to the development of PBCA-MB as pharmaceutical products for preclinical and clinical applications.

## 1. Introduction

Ultrasound (US) imaging is frequently employed in clinical practice because of its non-invasive nature, easy handling, low cost, real-time feedback, and broad applicability. Microbubbles (MB) extend the range of US applications towards functional and molecular imaging of (patho-) physiological phenomena in cardiovascular diseases and cancer [1,2]. MB are 1–5 µm-sized gas-filled vesicles, which are shell-stabilized by lipids, proteins, or polymers. Advances in MB formulation and functionalization have enabled the incorporation of various targeting ligands, imaging agents, and drug molecules, expanding their applicability towards multimodality imaging and direct and indirect drug delivery [3,4]. The suitability of different MB formulations for imaging and drug delivery is largely based on their shell composition (lipid-based soft MB vs. polymer-based hard MB). Hard-shelled polymeric MB, including poly(*n*-butyl cyanoacrylate) (PBCA) MB, are generally considered to be favorable for multimodality imaging and drug delivery [3,4,5].

PBCA-MB have been explored for various imaging and drug delivery purposes [6,7,8]. PBCA is a biodegradable polymer and is FDA-approved as a surgical superglue for wound closing [9,10,11,12]. The shell of PBCA-MB is composed of relatively small-sized polymer chains, with an average molecular weight (Mn) of 4 kDa and with more than 99% of the chains below 40 kDa. The diameter of PBCA-MB typically is around 2 µm, and the shell thickness can be varied from ~50 to ~300 nm [13]. This relatively thick shell enables stable encapsulation of air, preventing diffusion into dispersion media, such as the blood upon i.v. administration [14]. In addition, the much thicker shell of PBCA-MB as compared to lipidic MB (~3–5 nm) allows for more efficient and more stable shell-loading with contrast agents, fluorescent dyes, and drug molecules [6,7,8].

To enable the commercial use of PBCA-MB, either for preclinical or for clinical applications, it is important to study and optimize key pharmaceutical properties, such as formulation stability and shelf-life. Lyophilization (freeze drying) is a water removal process based on sublimation under vacuum conditions without the need to apply heat. Lyophilization dramatically increases the shelf-life of MB and potentially allows for long-term storage at room temperature. In addition, from a logistic point of view, lyophilization substantially reduces product weight and volume, making it more suitable for packaging, shipping, and storing. Because of this, establishing methods to lyophilize PBCA-MB are considered very valuable for product development.

**Figure 1:**
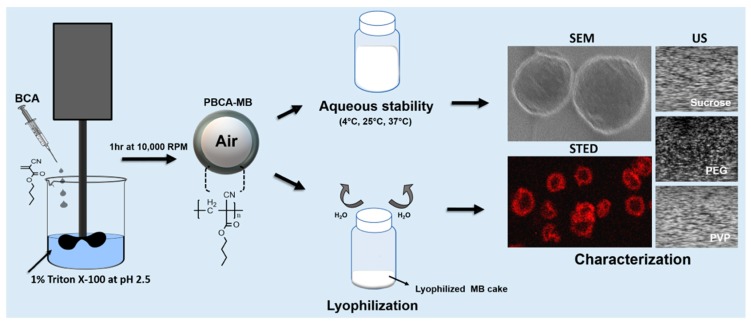
Study design. Poly(*n*-butyl cyanoacrylate) microbubbles (PBCA-MB) were synthesized via anionic polymerization of *n*-butyl-cyanoacrylate (BCA) at pH 2.5. After MB preparation the aqueous suspension of MB were stored at 4, 25, and 37 °C and the change in their physical properties were monitored over the duration of 20 weeks with techniques like Scanning electron microscope (SEM), stimulated emission depletion (STED) microscopy, and ultrasound (US). The aqueous MB suspension was also lyophilized. Furthermore, the lyophilizates were analyzed with the same techniques.

We here set out to systematically study the stability, shelf-life, and lyophilization of PBCA-MB (Figure 1). This was done by storing them for up to 20 weeks at 4 °C, 25 °C, and 37 °C, and monitoring changes in their concentration and physicochemical properties (size, acoustic signal generation). Rhodamine-containing PBCA-MB were also employed, as a model formulation for a contrast agent/drug-loaded MB. We evaluated the feasibility of lyophilizing PBCA-MB, using the commonly employed cryoprotectants glucose, sucrose, PVP, and PEG (Figure 1). Our findings show that PBCA-MB are stable at 4 °C and 25 °C for up to 20 weeks, in terms of concentration, size, and acoustic properties. Sucrose and PVP were found to be suitable cryoprotectants for freeze-drying both standard and dye-loaded PBCA-MB, providing a basis for the pharmaceutical development of lyophilized PBCA-MB for future preclinical and clinical applications.

## 2. Materials and Methods

### 2.1. Materials

Sucrose, glucose, PVP 40k, PEG-2k, Rhodamine-B, Triton-X, and gelatin were purchased from Merck (Darmstadt, Germany). DMSO was obtained from Carl Roth (Karlsruhe, Germany). *N*-butyl-cyanoacrylate (BCA) was purchased from Special Polymers (Sofia, Bulgaria). All the materials were analytical grade and used without purification.

### 2.2. Microbubble Synthesis

PBCA-MB were synthesized based on anionic-emulsion polymerization in the presence of hydroxyl ions, as described previously [13]. Briefly, 3 mL of BCA was added drop-wise to 300 mL aqueous solution containing 1% of Triton-X at pH 2.5. This mixture was emulsified at 10,000 RPM for 1 h at room temperature, using an Ultra-Turrax T-50 basic (IKA Werke, Staufen, Germany). The resulting solution was centrifuged at 46 G for 20 min and washed 4–5 times with 0.02% (*v*/*v* %) aqueous solution of Triton-X (pH~7) to remove non-MB-associated polymer fragments. The final formulations were stored in 50 mL of 0.02% (*v*/*v* %) Triton-X solution (pH = 7) and are referred to as the control batch throughout the manuscript.

### 2.3. Synthesis of Rhodamine-B Loaded Microbubbles

Rhodamine-B loaded MB were prepared based on the previously established “two-step” loading method [6,8]. To this end, 1 mg of rhodamine-B was added to 10 mL of pre-formed MB suspension under continuous stirring at 400 RPM for 4 h. Samples were washed several times with 0.02% aq. solution of triton-X (pH = 7) until the subnatant was free of residual dye. Rhodamine loading was analyzed using a microplate reader TECAN Infinite M200 Pro (TECAN group Ltd., Männedorf, Switzerland).

### 2.4. Analysis of MB Size and Concentration

Control and rhodamine-B loaded MB were characterized regarding their size and concentration using a Multisizer-4 (Beckman Coulter GmbH, Krefeld, Germany). A 30 µm capillary tube was used with a flow rate of 100 µL/min to measure MB size in the range of 0.6 to 18 µm. To this end, 2 µL of MB solution was mixed with 20 mL of ISOTON^®^ II (Beckman Coulter GmbH, Krefeld, Germany), prior to the analysis. Measurements were obtained in triplicate and results are presented as average ± SD.

### 2.5. Analysis of Storage Stability

PBCA-MB were stored at 4 °C, 25 °C, and 37 °C, in triplicates, at a concentration of 1 × 10^9^ MB/mL in screw-cap glass vials. The size and concentration of the MB were measured weekly over a period of 20 weeks. Similarly, rhodamine-B loaded PBCA-MB samples were analyzed up until 4 weeks, looking particularly at dye retention. All measurements were performed in triplicate and results are presented as average ± SD.

### 2.6. Ultrasound Imaging of PBCA-MB

MB kept at 4 °C, 25 °C, and 37 °C were subjected to US imaging, up until 20 weeks, to analyze the effect of storage on US signal generation properties. US imaging was performed using tissue-mimicking gelatin phantoms and a VisualSonics VEVO2100 US system (FUJIFILM VisualSonics, Amsterdam, The Netherlands) equipped with a MS 250 linear-array based transducer (25 MHz central frequency). Approximately 6 × 10^4^ MB were mixed with 4.5 mL of 2% *w*/*v* gelatin and embedded in 10% gelatin bulk. The US transducer was fixed vertically to the gelatin phantom at a focus depth of 11 mm. Imaging was performed in non-linear contrast mode at 18 MHz frequency and 4% power (MI 0.2) and 104 frames were recorded. For quantification, a region-of-interest (ROI) of ~1.4 mm^2^ was drawn at the focus point of the transducer, from which the contrast intensity of the incubated MB samples was analyzed using Vevo LAB software. The obtained contrast intensity was compared with freshly prepared control MB.

### 2.7. Lyophilization

Lyophilization/freeze drying of PBCA-MB was performed using different cryoprotectants, including the sugars sucrose and glucose, and the polymers PVP and PEG. For this, 5% *w*/*v* solutions of the mentioned cytoprotectants were mixed with PBCA-MB and Rho-PBCA-MB at a dose of 3.5 × 10^9^ MB/mL. Samples were gently mixed, snap-frozen in liquid nitrogen, and, subsequently, freeze-dried using bench-top lyophilizer (CHRIST Alpha 2-4 LD plus, Osterode am Harz, Germany) for 24 h at a vacuum pressure of 0.1 mbar and at a condenser temperature above –80 °C. The dried samples were re-dispersed in a 0.02% Triton-X (*v*/*v* %) aqueous solution.

### 2.8. Scanning Electron Microscopy

Cryo-scanning electron microscopy was used to investigate the effect of the four cytoprotectants on the size, shell thickness, and surface morphology of PBCA-MB. Measurements were obtained using a FE-SEM 4800 (Hitachi, Krefeld, Germany) having an Alto 2500 Cryo-Gatan unit (Gatan GmbH, Munich, Germany). Standard and rhodamine-B loaded MB were capped onto the sample holder and frozen in liquid nitrogen. The sample holder was then inserted into the preparation chamber and the MB droplets were cut using a scalpel. Readings were taken at 1 kV and 2 µA.

### 2.9. US Imaging of Lyophilized MB

The MB cake obtained after lyophilization was immediately re-suspended in 1 mL of 0.02% of Triton-X (*v*/*v*) and subjected to US imaging immediately afterwards. Approximately, 3 × 10^4^ MB were mixed with 4.5 mL of 2% (*w*/*v*) gelatin and embedded in 10% gelatin. Imaging was performed in non-linear contrast mode at 18 MHz frequency, 4% power, and 104 frames (18 frames per sec) were recorded. For quantification, a region-of-interest (ROI) of ~1.4 mm^2^ was then placed at the focus point of the transducer, from which the contrast intensity of the different MB formulations was analyzed using Vevo LAB software (FUJIFILM VisualSonics, Amsterdam, The Netherlands) and compared with non-lyophilized control samples.

### 2.10. Fluorescence Measurements and STED Microscopy

The concentration of rhodamine-B in rho-PBCA-MB was analyzed based on standard excitation/emission calibration curve at 540 and 577 nm using TECAN Infinite M200 Pro (TECAN group ltd., Männedorf, Switzerland). To avoid fluorescence quenching of rhodamine-B, rho-PBCA-MB were stored in dark chambers and vials were covered with aluminum foil. Fluorescence analysis of rhodamine-B loaded PBCA-MB was performed right after obtaining the lyophilized cake via Leica TCS SP8 X automated inverted confocal and stimulated emission depletion (STED) microscope equipped with a plan-apochromat 100×/1.40 oil-immersion objective. Image analysis was done using Image J software (ImageJ, Bethesda, USA). For quantification, MB were randomly selected in five different regions in the microscopy images. The obtained mean fluorescence intensity values were normalized with image background signal, size and number of microbubbles.

### 2.11. Statistical Analysis

Statistical analysis was performed using GraphPad Prism 8 (GraphPad Software Inc., San Diego, CA, USA). One-way ANOVA with Geisser-Greenhouse correction and Dunnett multiple comparisons test was done for all the analysis. *p-*values of less than 0.05 were considered to be statistically significant.

## 3. Results

### 3.1. Effect of Shelf Temperature on the Size and Concentration of PBCA-MB Overtime

**Figure 2:**
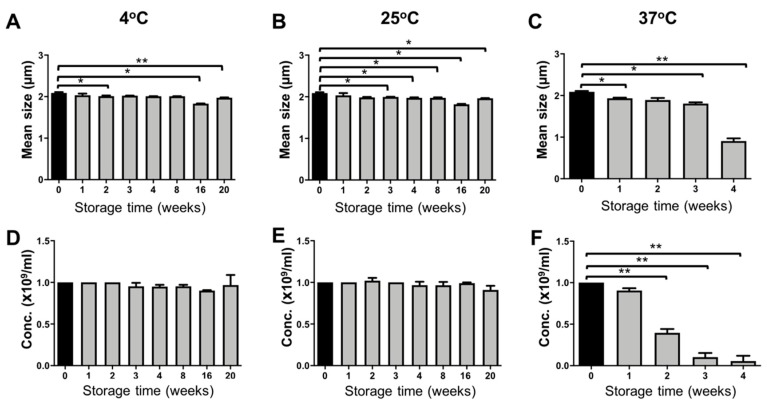
Storage stability of PBCA microbubbles. The average size and concentration (number of MB ×10^9^ per mL) of PBCA-MB stored at 4 °C, 25 °C, and 37 °C were monitored for up to 20 weeks (**A**–**F**). For samples kept at 37 °C, significant decreases in size and concentration were observed from week 4 and week 2 onwards, respectively (**C**, **F**). Values represent average ± SD of three different batches, measured in triplicates. * and ** indicates *p* < 0.05 and *p* < 0.005, respectively.

PBCA-MB were prepared via anionic emulsion polymerization of BCA (butyl cyanoacrylate) monomers in presence of hydroxyl ions at pH 2.5 (Figure 1) [13]. After MB size isolation via centrifugation and flotation, the average diameter of the MB was approximately two microns, with narrow size distribution. The size and concentration measurements for PBCA-MB stored at three different temperatures (4 °C, 25 °C, and 37 °C) were done weekly for 20 weeks, using a coulter counter. As illustrated in Figure 2, significant but only very minor changes in the size and concentration were observed for MB stored at 4 °C and 25 °C. This good stability results from the relatively rigid PBCA shell, which prevents the escape of air in the dispersion medium [1,15]. Conversely, at 37 °C, a decrease in MB size was observed already after one week of storage, and this dropped strongly to half of its initial value in the fourth week (Figure 2C). In line with this, an even stronger drop in the concentration of the MB was observed from the second week onwards. At week three and four, more than 80% and more than 90% of the MB had disintegrated (Figure 2F). At increasing temperatures, the amount of dissolved gas in aqueous media decreases more rapidly, which indirectly promotes the diffusion of gas from the MB core into the dispersion medium, leading to MB shrinkage and collapse [16]. These results demonstrate that PBCA-MB are properly stable for up to 20 weeks at 4 °C and 25 °C, and that increasing the temperature to 37 °C results in relatively rapid disintegration.

### 3.2. Effect of Storage Time and Temperature on the Acoustic Properties of PBCA-MB

**Figure 3:**
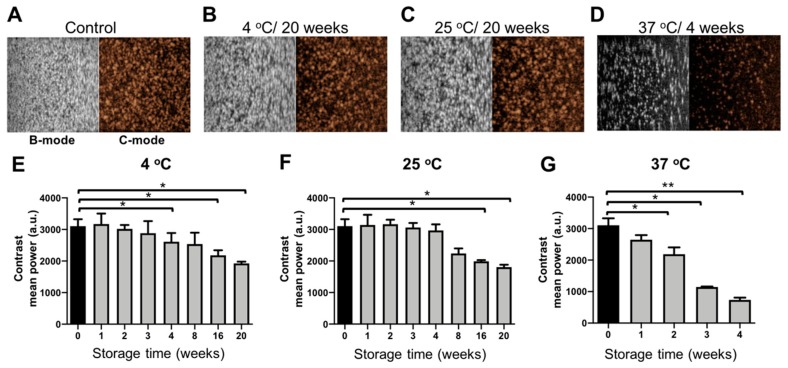
Acoustic properties of PBCA microbubbles upon storage. Representative bright mode (B-mode) and contrast mode (C-mode) US images of freshly prepared control PBCA-MB and PBCA-MB stored at 4 °C, 25 °C, and 37 °C for up to 20 weeks (**A**–**D**). Quantification of the US images revealed no significant differences in backscatter signals when MB were stored at 4 °C and 25 °C (**E**,**F**). For samples stored at 37 °C, the acoustic signals decreased significantly from week 1 onwards (**G**). Values represent average ± SD of three different batches, measured in triplicates. * and ** indicates *p* < 0.05 and *p* < 0.005, respectively.

It is known that the acoustic performance of MB is dependent on (1) the type of gas in the core; (2) under/over-saturation of the gas in the aqueous medium; and (3) type of the encapsulating shell, which provides a permeation barrier for gas escape [17,18,19,20,21,22]. Therefore, the acoustic signal generation properties of PBCA-MB stored at 4, 25, and 37 °C were analyzed weekly for up to 20 weeks, using gelatin phantoms and an in vitro ultrasound setup. Time intensity curve analysis revealed a gradual decrease in US contrast between fresh MB and MB stored at 4 °C and 25 °C over the period of 20 weeks (Figure 3E,F). For MB stored at 4 °C, a significant decrease in contrast intensity was observed from week four onwards, whereas in case of MB stored at 25 °C, this was observed from week eight onwards.

Our PBCA-MB formulations contain air in their core, which helps to prevent the dissolution of the gaseous phase from the MB core due to a concentration gradient, as commonly observed for commercially available lipid-based MB containing heavy gases. The upper gaseous phase in our sample vials is naturally saturated with air and thereby aids in conserving the air volume inside the MB core for prolonged periods of time. In addition to this, the high hydrophobicity of the PBCA shell generates permeation resistance to air/gas escape into the aqueous medium [6,23,24]. Furthermore, the intrinsically very thick shell of PBCA-MB (typically 50 to 300 nm) constitutes a more formidable permeation barrier for air/gas escape as compared to lipid-based MB, which typically have very thin shells (approx. 3–5 nm) [1,15]. This is justified by the partition-diffusion theory, which predicts a linear dependence between shell resistance to mass transfer and shell thickness [23]. Consequently, the slow dissolution of air from the PBCA-MB core and the presence of a thick PBCA shell together help to preserve the acoustic properties of PBCA-MB in aqueous solution at 4 °C and 25 °C up to three and four weeks.

On the contrary, when the MB samples were stored at 37 °C, the acoustic contrast decreased over time (Figure 3G). This decrease is already evident after a week of storage, and at the end of week three more than 50% of acoustic backscattering ability has disappeared. As detailed above, this is likely due to disintegration of MB, which is line with the coulter results. Moreover, incubation at 37 °C results in the selection of more stable MB, with a stiffer shell, showing less oscillating behavior. The decrease in MB size at 37 °C is caused by enhanced diffusion of air from the MB core into the aqueous medium at this temperature [16]. Various reports in the literature corroborate the notion that an increase in temperature decreases MB size and US signal generation properties [19,25,26,27]. The decrease in US contrast after one week of storage at 37 °C also correlates with the coulter counter measurements, showing a similar decrease in MB size and concentration within one week.

### 3.3. Effects of Lyophilization on the Size, Concentration, and Morphology of PBCA-MB

**Figure 4:**
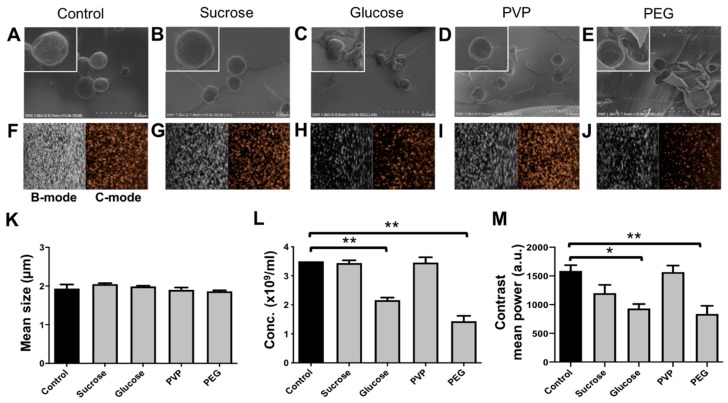
Lyophilization of PBCA microbubbles with different cryoprotectants. Representative SEM imaging (**A**–**E**) of freshly prepared control PBCA-MB, and PBCA-MB lyophilized with 5% sucrose, glucose, polyvinylpyrrolidone (PVP), and polyethylene glycol (PEG). Representative bright mode (B-mode) and contrast mode (C-mode) US images of non-lyophilized control PBCA-MB and PBCA-MB lyophilized with 5% sucrose, glucose PVP, and PEG (**F**–**J**). The impact of lyophilization on MB size, concentration, and acoustic signal generation as compared with non-lyophilized control MB show that sucrose and PVP are suitable cryoprotectants for freeze-drying PBCA-MB (**K**–**M**). Values represent average ± SD of three different batches, measured in triplicates. * and ** indicates *p* < 0.05 and *p* < 0.005, respectively.

The effects of lyophilization (freeze-drying) on size and concentration of PBCA-MB cryoprotected by mono- and disaccharides of glucose and sucrose and the polymers PVP and PEG were analyzed using coulter counter measurements. In addition, SEM was employed, for structural analysis of lyophilized PBCA-MB. The coulter counter measurements in Figure 4K indicate no significant changes in the size of PBCA-MB lyophilized with different cryoprotectants when compared to freshly prepared PBCA-MB. This suggests that the size of lyophilized microbubbles upon reconstitution is unaffected by freeze-drying. Furthermore, no change in concentration was observed for PBCA-MB cryoprotected with 5% sucrose and 5% PVP (Figure 4L). Moreover, MB lyophilized with 5% sucrose and 5% PVP and, subsequently, reconstituted and stored for up to five weeks at 25 °C were stable in terms of size and concentration (Figure S1E,F). Conversely, the concentration of PBCA-MB lyophilized using 5% glucose and 5% PEG significantly dropped as compared to the starting formulation (Figure 4L).

The scanning electron microscopy (SEM) images in Figure 4A–E illustrate the morphology of freshly prepared PBCA-MB and PBCA-MB lyophilized using the four different cryoprotectants. The size, shape, and surface morphology of sucrose- and PVP-protected PBCA-MB were identical to those of non-lyophilized PBCA-MB. In case of glucose- and PEG-lyophilized MB, on the other hand, cracks in the MB shell, uneven shapes, and polymer fragments were observed, most likely due to MB disintegration and coalescence during freeze-drying. Together, these results demonstrate that sucrose and PVP are useful cryoprotectants for preparing lyophilized PBCA-MB formulations.

At the lyophilization settings employed in this study, both glucose and PEG are found to be unsuitable as cryoprotectants for the freeze-drying of PBCA-MB. This is expected to be due to the low collapse temperature and the low glass transition temperature of glucose and PEG (i.e., −41 °C and −62.3 °C, respectively), resulting in disintegration and/or coalescence of PBCA-MB during freeze-drying [28,29]. For efficient lyophilization, freeze-drying should be carried out at temperatures below the collapse temperature of the product [28]. Due to limitations of our current lyophilizer setup, such low temperatures could not be achieved. In future studies, we will verify and expand these analyses using more specialized equipment. With the device and at the settings employed here, freeze-drying worked well for sucrose and PVP, which have higher collapse temperature (−31 °C and −24 °C) [28]. In general, higher collapse temperatures are more favorable for freeze-drying, as it allows the drying process to be carried out at a higher temperature, thereby reducing the drying time and increasing the cost-efficiency [30]. Taken together, based on the settings evaluated and result obtained thus far, we conclude that PVP and sucrose are suitable cryoprotectants for lyophilizing PBCA-based MB.

### 3.4. Effects of Lyophilization on the Acoustic Properties of PBCA-MB

We next set out to evaluate if PBCA-MB lyophilized using sucrose, glucose, PVP, and PEG as cryoprotectants are able to retain good US signal generation properties. MB samples lyophilized using 5% of the different cryoprotectants were reconstituted in 0.02% (*v*/*v* %) Triton-X aqueous solution and embedded in gelatin phantoms. Quantification of US time intensity time curves showed only minor difference in contrast generation between samples non-lyophilized MB and MB lyophilized using 5% sucrose whereas for MB lyophilized with 5% PVP showed no difference at all (Figure 4M). Moreover, reconstituted MB lyophilized with 5% sucrose and 5% PVP were stable in terms of acoustic signal generation for up to five weeks when stored at 25 °C (Figure S1G) Conversely, MB lyophilized using 5% PEG and 5% glucose produced 47% and 41% less US contrast, respectively. It should be noted that for all four conditions, equal numbers of (lyophilized and reconstituted) MB were embedded in the gelatin phantoms. Therefore, the decrease in contrast does not result from variability in MB concentration, but should be attributed to changes in their mechanical (e.g., surface tension) [31] and/or morphological properties (e.g., spherical shape) during freeze-drying and gelatin phantom preparation. Changes in morphological properties can be seen in SEM images of glucose and PEG in the upper panels in Figure 4, showing cracked morphology, uneven geometry, and deviation from spherical shape. Spherical shape is thermodynamically more stable and offers minimum surface tension at the bubble-air/water interface, conveying higher stability against MB coalescence or rupture.

### 3.5. Effect of Lyophilization on the Size, Concentration, and Morphology of Dye-Loaded MB

**Figure 5:**
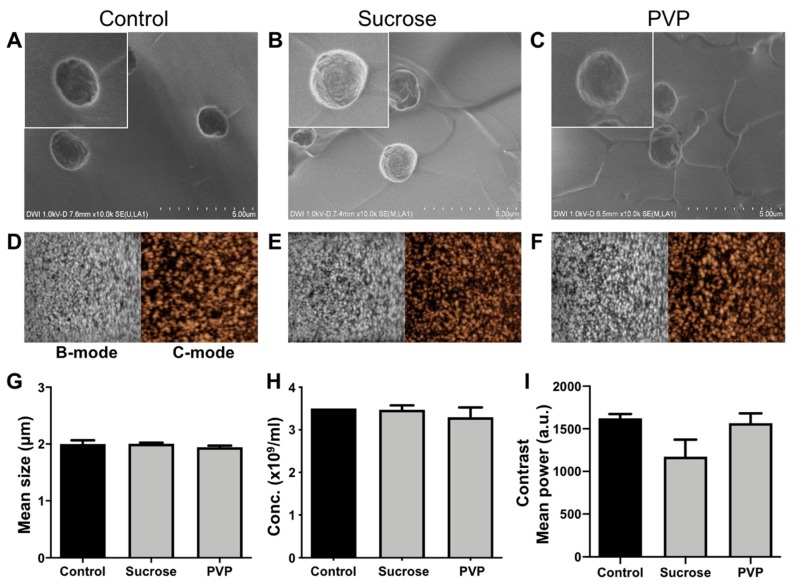
Characterization of dye-loaded PBCA-MB before and after lyophilization. Representative SEM images of non-lyophilized control rhodamine-B (Rho) PBCA-MB versus Rho-PBCA-MB lyophilized using 5% sucrose and 5% PVP indicating no changes in surface morphology (**A**–**C**). Representative bright mode (B-mode) and contrast mode (C-mode) US images of non-lyophilized control PBCA-MB versus PBCA-MB lyophilized with 5% sucrose and PVP (**D**–**F**). Lyophilization did not affect the size and concentration of Rho-PBCA-MB as compared to fresh non-lyophilized control Rho-PBCA-MB samples (**G**,**H**). Quantification of the US backscatter showed only very slight changes in US signal generation between non-lyophilized and Rho-PBCA-MB lyophilized with 5% sucrose whereas no change in case of Rho-PBCA-MB lyophilized with 5% PVP (**I**). Values represent average ± SD of three different batches, measured in triplicates.

Rhodamine-B (Rho) can be loaded into PBCA-MB as a fluorophore to enable multimodal optical/US imaging, as well as a model drug to explore the possibility of doing US- and MB-mediated drug delivery. In addition to the above studies, we analyzed how the loading of Rho in MB affects the lyophilization process, and also how fluorophore-/model drug-retention is affected by lyophilization. In these experiments, we only used sucrose and PVP as cryoprotectants, as they clearly outperformed glucose and PEG in our freeze-drying setup. The loading efficiency of rhodamine-B in PBCA was determined by spectroscopy and was found to be 2.14 ± 0.11%. Size and concentration analysis based on coulter counter measurements did not show any significant changes for lyophilized vs. non-lyophilized Rho-PBCA-MB (Figure 5G,H). In line with this, SEM analysis did not show any difference in the size and surface morphology of 5% sucrose-Rho-PBCA-MB and 5% PVP-Rho-PBCA-MB as compared to non-lyophilized control Rho-PBCA-MB and PBCA-MB (Figure 5A–C and Figure 4A). These findings indicate that the presence of rhodamine-B in the polymeric shell does not influence the lyophilization of PBCA-MB when employing the cryoprotectants sucrose and PVP.

### 3.6. Effects of Lyophilization on the Acoustic Properties of Dye-Loaded PBCA-MB

Rhodamine-loaded PBCA-MB lyophilized using 5% PVP and 5% sucrose were reconstituted and embedded in gelatin phantoms for US imaging as shown in representative B-mode and C-mode US images (Figure 5D–F). Analysis of the US phantom images showed only slight differences in the contrast enhancement between non-lyophilized controls and Rho-PBCA-MB lyophilized using 5% sucrose and 5% PVP (Figure 5I). As exemplified in the SEM images in Figure 5A–C, lyophilized and reconstituted Rho-PBCA-MB display well-defined spherical morphology, similar to non-lyophilized controls. This indicates that both PVP and sucrose are suitable cryoprotectants for preventing MB disintegration also in the presence of Rho in the MB shell. Together, these results demonstrate that Rho-PBCA-MB can be efficiently lyophilized and re-constituted, ensuring good US contrast and dye (model drug) retention.

### 3.7. Effect of Lyophilization on Dye Retention in PBCA-MB

**Figure 6:**
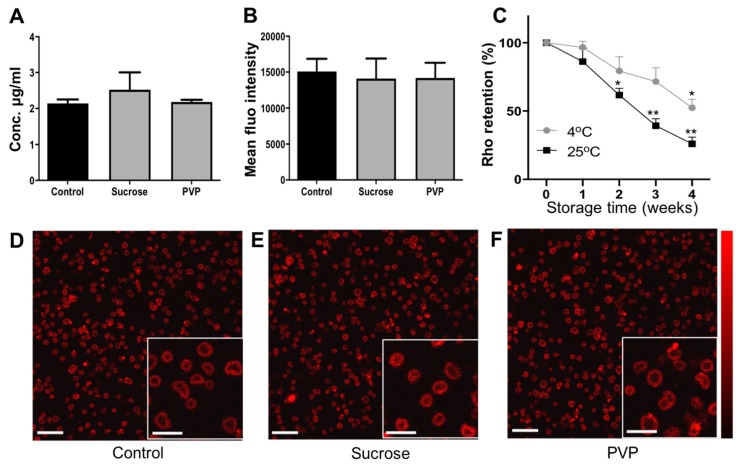
Dye retention in lyophilized PBCA-MB. No change in rhodamine-B (Rho) concentration was detected post lyophilization of Rho-PBCA-MB when compared to the initial concentrations (**A**). Quantification of STED microscopy images confirmed no significant change in the mean fluorescence intensity of lyophilized Rho-PBCA-MB compared to that of non-lyophilized control PBCA-MB (**B**). Quantification of Rho retention in non-lyophilized PBCA-MB showed a gradual decrease in rhodamine amounts from the first week of incubation at 4 °C and 25 °C onwards (**C**). Representative STED images of non-lyophilized control Rho-PBCA-MB and Rho-PBCA-MB lyophilized using sucrose and PVP (**D**–**F**). Scale bars represent 10 μm for the large image and 5 μm for the insets. Values represent average ± SD of three different batches, measured in triplicates. * and ** indicates *p* < 0.05 and *p* < 0.005, respectively.

The effect of lyophilization on rhodamine-B retention in PBCA-MB was investigated using fluorescence analysis on a TECAN plate reader as well as STED microscopy. Compared to control Rho-PBCA-MB, TECAN-based fluorescence measurements show no change in the concentration and fluorescence intensities of rhodamine-B in Rho-PBCA-MB cryoprotected with sucrose and PVP as compared to controls (Figure 6A). This was confirmed by STED microscopy, visualizing and analyzing the fluorescence intensity of non-lyophilized and lyophilized Rho-PBCA-MB. The STED microscopy images clearly show the localization of fluorescence signal in the polymeric shell of control and lyophilized Rho-PBCA-MBs (Figure 6D–F). Image analysis indicated no change in the fluorescence intensity of the Rho-PBCA-MB cryoprotected with 5% sucrose and 5% PVP as compared to non-lyophilized Rho-PBCA-MB (Figure 6B), exemplifying that fluorophores (and model drugs) can be efficiently retained within the MB shell upon lyophilization.

When Rho-PBCA-MB are stored over weeks at 4 °C and 25 °C, however, a gradual decline in the concentration of rhodamine-B was observed in TECAN measurements (Figure 6C). At the fourth week, the rhodamine-B concentration significantly dropped to values below 52% and 25% of the initial concentration at 4 °C and 25 °C, respectively. This indicates significant leakage over time of rhodamine-B into to aqueous medium, due to the relatively weak hydrophobic interactions between rhodamine-B and the PBCA-based MB shell. This, however, is not the case with lyophilized Rho-PBCA-MB cake as leakage of rhodamine cannot take place due to the removal of aqueous phase during freeze-drying. Overall, these results demonstrate that the lyophilization of Rho-PBCA-MB can provide long term storage stability not only in terms of size and concentration, but also fluorophore (model drug) retention.

## 4. Conclusions

Our findings demonstrate proper stability of PBCA-based polymeric MB at 4 °C and 25 °C over a period of three to four weeks, in terms of size, concentration, and acoustic properties. At 37 °C, PBCA-MB were stable for only one week. We furthermore show that prolonged stability of PBCA-MB can be achieved using lyophilization (freeze-drying). Lyophilization experiments showed that sucrose and PVP are superior to glucose and PEG in term of cryoprotecting PBCA-MB and rhodamine-B loaded PBCA-MB. Freeze-drying prevented rhodamine-B leakage from PBCA-MB, which inevitably occurred when PBCA-MB are kept in aqueous suspension. Together, these results exemplify the robustness of PBCA-based polymeric MB, and they are valuable for moving PBCA-MB formulations forward as pharmaceutical products for preclinical and clinical applications.

## Supplementary Materials

The following are available online at https://www.mdpi.com/1999-4923/11/9/433/s1, Figure S1: Characterization of lyophilized PBCA-MB upon reconstitution and storage.
