# Supplementary material for: Shelf-Life Evaluation and Lyophilization of PBCA-Based Polymeric Microbubbles
Source: Pharmaceutics. 2019 Aug 26;11(9):433. doi: 10.3390/pharmaceutics11090433 (PMC6781551; doi:10.3390/pharmaceutics11090433)
Supplement: Supplementary file 1 [file pharmaceutics-11-00433-s001.pdf]

pharmaceutics

MDPI

Supplemntary Materials: Shelf-life evaluation and lyophilization of PBCA-based polymeric microbubbles

Tarun Ojha, Vertika Pathak, Natascha Drude, Marek Weiler, Dirk Rommel, Stephan Rütten, Bertram Geinitz, Mies J. van Steenbergen, Gert Storm, Fabian Kiessling and Twan Lammers

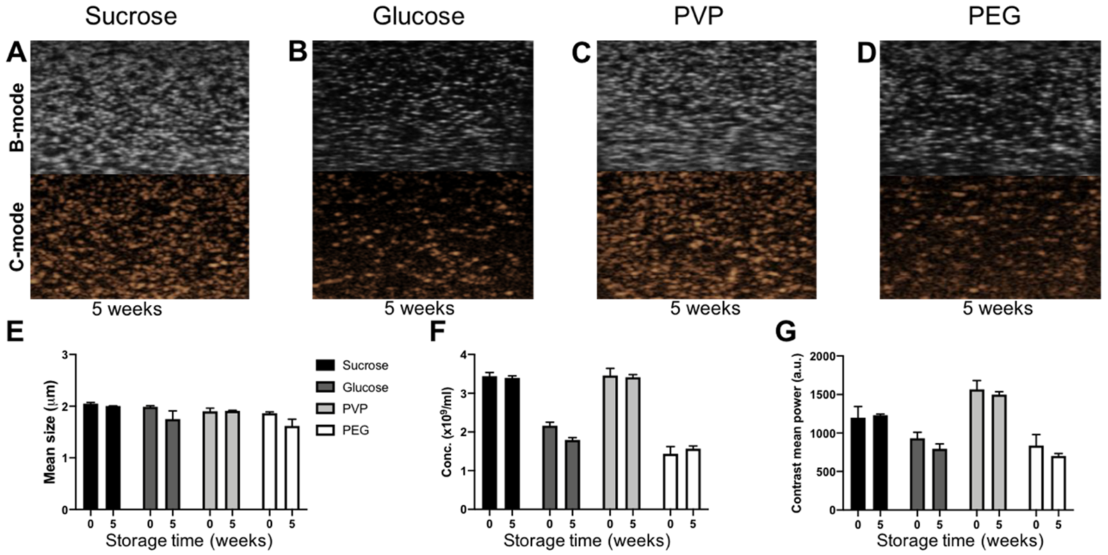

Figure S1. Characterization of lyophilized PBCA-MB upon reconstitution and storage. Representative bright mode (B-mode) and contrast mode (C-mode) US images (**A**–**D**) of reconstituted PBCA-MB upon initial lyophilization with 5% sucrose, glucose, PVP and PEG and subsequent storage at 25 °C for 5 weeks. The average size and concentration of lyophilized and reconstituted PBCA-MB monitored (**E**–**F**). A marginal reduction in size and concentration of reconstituted was observed for MB lyophilized with 5% glucose and PEG after 5 weeks of storage at 25 °C whereas no change was seen for MB lyophilized with 5% sucrose and PVP. Similarly, quantification of the ultrasound images revealed a marginal decrease in backscatter signals of MB lyophilized with 5% glucose and PEG after 5 weeks of storage at 25 °C (**G**) whereas no change was observed for MB lyophilized with 5% sucrose and PVP. Values represent average ± SD of three different batches, measured in triplicates.
